# Ophthalmia Neonatorum

**Published:** 1891-12

**Authors:** Frank Trester Smith

**Affiliations:** Prof. of Diseases of the Eye, Chattanooga Medical College, Chattanooga, Tenn.


### OPHTHALMIA NEONATORUM.

By FRANK TRESTER SMITH, A. M., M. D.,
Prof. of Diseases of the Eye, Chattanooga Medical College, Chattanooga, Tenn.

The importance of the subject is suggested by three facts:

1st. Of the number of blind in our asylums a large per cent. of the blindness is produced by this cause.

2d. The increase in the number of blind in the United States has been increasing in a greater ratio than the population.

3d. Blindness from this cause is entirely preventable.

So important is this subject regarded in Europe that many of those countries have special legislation on the subject. This legislation generally provides that at the time the birth is registered a card is given the mother cautioning her about the danger of any discharge from the eye. Some laws require the midwives to report any redness or discharge.

To Dr. Howe, of New York, we are indebted [illegible] alling attention to the frequency of blindness and the facts concerning its increase.

Since the introduction of Credé's method the percentage of cases has been materially decreased in large lying-in hospitals. In his own service from 7.85 to .31 per cent.

The cause is generally conceded to be the introduction of the gonococcus at or soon after the time of birth. The prevention is an easy matter, and is of most importance to us.

During my service at the Emigrant Hospital, Ward's Island, New York, in the lying-in department, the vagina was douched with a sublimate solution (1:2000). As soon as the child was born the eyes were washed with a solution of common salt in water, about ten per cent. As a result, ophthalmia neonatorum was unknown in that institution during my service.

One of the great difficulties here is that many practitioners

*Read before the Chattanooga Medical Society.

will never see a case in the course of a large practice covering many years, and it is admitted that the danger is small in private practice, but as long as there is any danger at all and the method of prevention so simple and so sure, would it not be well to adopt these measures in all cases? Credé's method is to instill into the conjunctival sac a few drops of a two per cent. solution of nitrate of silver as soon as the child is born. This solution sometimes produces a considerable reaction, so that cold applications were used to lessen the inflammation, but this was rarely the case, and this inflammation never resulted disastrously. An application that would produce less reaction, and one that probably is as efficient, is the use of a solution of corrosive sublimate (1:3000) used in the same manner.

The diagnosis of these cases is easy, and is made from the secretion. But know more than this. It is of importance to know whether the cornea is involved or not, both for the treatment and for the prognosis. But right here we must not be too curious, for if we endeavor to inspect the cornea with a struggling child pressing the lids firmly together, we may exert enough pressure on the ball to break through the floor of an ulcer which has eaten its way to the membrane of Descemet. It is better to be satisfied with an unfinished diagnosis and to treat the case as though there were corneal complications present, than to take any unnecessary risks.

The prognosis depends on the condition of the cornea. The danger is only from the involvement of the structure. As long as we can keep this free from ulceration we have no fear of the result.

The treatment is one in which the authorities disagree in some particulars. We speak of the treatment under the following heads:

1st. Cleanliness. On this all are agreed. It is best accomplished by the use of a solution of boracic acid gr. x ad. ℥j., with which the discharge is to be washed from the eye as often as it accumulates, whether it be every ten minutes or every hour.

2d. The use of heat or cold. Here there is some difference of opinion, some teaching to use whichever feels the most comfortable to the patient, while others advocate the use of cold or heat exclusively.

When we consider the cause of the disease and remember that the cocci can be prevented from multiplying by a low temperature; when we know that we can produce this temperature in the conjunctival sac by actual observation; when we consider further the rapid changes, often destructive in character, produced by heat; and finally the fact that as a rule heat is not as well borne in these cases, we must conclude not only that cold applications are most rational, but that they are the best for the patien

Cold is to be applied in the following manner: From twelve to fifteen pieces of white linen cloth two inches square are folded twice, slightly moistened and placed on a cake of ice alongside the bed. One of these is placed on the closed eyelids and allowed to remain there until it begins to get warm, when it is changed for a cold piece, and this is kept up, so that we have a continuous application of cold without any pressure, as we would have from the use of an ice bag, however small. This plan necessitates the employment of at least two nurses, for the applications are to be kept up night and day. To prevent a silght irritation of the skin which we sometimes have, we may rub a little vaseline on the outside of the lids and the surrounding skin. This employment of cold is not intended as an adjuvant to the treatment, but is to be relied on as the principal part of the treatment.

The 3d item in the treatment refers to the use of nitrate of silver. In the use of this drug oculists differ not only as to the stage of the disease when it is to be used, but also as to the strength of the solution. The practice at the New York Ophthalmic and Aural Institute is to use it in solution no stronger than 2 per cent. (gr. x ad. ℥i). Some use much stronger solutions. The objection to this practice is that where there is an ulcer of the cornea, in some cases the silver has a bad effect. I remember in one case where the solution seemed to penetrate into the middle layers of the cornea, while the external layers seemed to be intact; it seemed to decompose this layer, at any rate that eye was lost. Knapp employs the following method to protect the cornea. The upper and lower lids are everted at the same time, and the upper lid is allowed to descend until it covers the ball; we can then apply the solution so that none of it comes in contact with the corneal tissue.

The solution is never used until the secretion is well established, until the discharge becomes somewhat profuse. Many apply it in the early stage, and rely on it as the main line of treatment, while here it is regarded as an adjuvant.

The complications are the corneal troubles that too often accompany this disease. What we most dread is an ulceration of cornea. The cornea may be involved in three ways: 1st, by continuity of inflammation; 2d, by a choking of tissue at its margin, shutting off the nutrition: 3d, by direct infection. This is probably the most frequent way in which the corneal complication takes place. The roughened lids brushing over the delicate epithelium produce abrasions through which the acrid secretions gain access to the proper substance of the cornea. The two causes combined tend to make the ulcers deeper, until the membrane of Descemet is reached, which is not liable to give way unless some violence is used. Should this membrane be broken, the cocci would gain access to the delicate structure within the ball and set up an inflammation that could hardly fail to destroy the eye. Oftener, however, the eye is destroyed by the inflammation involving the whole of the cornea, which is then replaced with a cicatricial tissue; as this is not transparent the eye is destroyed for all practical purposes. The anterior part of the ball may shrink (phthisis bulbi). The opacity may not be so extensive as described. It varies from a faint nebula that can hardly be diagnosed, but may not interfere seriously with vision, to the total opacity just described. Atrop. sulph. (gr. jv ad. ℥i, is to be used for any acute corneal complication.

The treatment of the opacities does not always receive the attention that it should. Persistent treatment will clear up many of them in a wonderful degree. Where this cannot be done the formation of an artificial pupil behind a clear part of the cornea is often indicated. Wherever there is any clear cornea and the patient has perception of light, he should have the benefit of an examination by an expert, for some that are now blind may be made to see.

As to the other eye in these cases, it should be protected. This can only be done by seeing that it is kept clean and that none of the dressings from the affected eye come in contact with it, directly or indirectly. We cannot seal up an eye to any advantage in an infant.

Those who are desirous of looking up this subject further are referred to an article by Weeks in the *New York Medical Record*, of July 24th, 1886.

***

*Discussion.*—DR. COBLEIGH said that in a long experience he had never seen a case of ophthalmia neonatorum, but had seen many cases of catarrhal ophthalmia from the use of soap.

DR. KUYKENDALL said that the disease was more frequent in the cities. The gonococcus was found in 65 to 75 per cent. of the cases that had been diagnosed by good clinicians as the disease in question. Chlorine water is the best in the early stage. Every case can be saved if seen before the cornea is involved. One of the best authorities, D. B. Roosa, does not use the nitrate of silver at all. The use of the bichloride every half hour, if the cornea is involved, (1:4000) is good practice. If we can see only the lower part of the cornea, we can judge as to whether it is inflamed or not. If in doubt, treat as if there were an ulcer, but don't use too much force in making the examination, for the reason given in the paper. The use of silver by Knapp's method is not effective, because it does not reach the cul-de-sac where it is most needed. In cases where there seemed complete opacity he had seen surprising results from the use of calomel, but it is difficult to get these cases to keep up the treatment for years.

DR. TRIPPE believed in the use of the silver nitrate in strong solutions, and related a case where the two per cent. solution had no effect, and he got no good result until he increased the strength to gr. xxx ad. ℥i.

DR. TOWNES said that the silver may decompose when it comes in contact with the tissues, by uniting with the albumen of the tissues and forming the albumate of silver. A better effect would be produced by cleansing the tissue first.

DR. RATHMELL asked Dr. Smith what he did for the pain in these cases and in similar cases for the adult.

DR. SMITH said that he gave anodynes when necessary to the adult, but in the infant we had no evidence that the disease was painful. Under no circumstances was cocaine to be used, as it had a tendency to interfere with the nutrition of the cornea. He wanted to emphasize the use of cold as the main reliance in the treatment, which he had probably not dwelt upon fully in the paper.

